# Phytochemical composition, antilipidemic and antihypercholestrolemic perspectives of Bael leaf extracts

**DOI:** 10.1186/s12944-018-0713-9

**Published:** 2018-04-03

**Authors:** Nosheen Asghar, Zarina Mushtaq, Muhammad Umair Arshad, Muhammad Imran, Rabia Shabir Ahmad, Syed Makhdoom Hussain

**Affiliations:** 1 Department of Food Science, Nutrition and Home Economics, Government College University, Faisalabad, Pakistan; 2 Institute of Home and Food Sciences, Faculty of Life Sciences, Government College University, Faisalabad, Pakistan; 3 Department of Zoology, Wildlife and Fisheries, Government College University Faisalabad, Faisalabad, Pakistan

**Keywords:** *Aegle marmelos*, Bael, Water extract, Alkaloids, Phenolics, Hypercholesterolemia, Hyperlipidemia

## Abstract

**Background:**

In recent times, focus on plant research has improved all over the world and essential parts of plants provide bioactive compounds in human diet. The bael (*Aegle marmelos*) has enormous traditional uses in the treatment of chronic diarrhea, dysentery, peptic ulcers and as a laxative. The main focus of this study was characterization of bael leaf extract for its bioactive constituents, antihypercholestrolemic and antilipidemic perspectives.

**Methods:**

After proximate composition of bael powder, the aqueous extract of bael leaf was used for phytochemical profiling (alkaloids, total phenolic content and total flavonoid content). Afterwards, normal rats group G_0_ was administrated basal diet while G_1_ and G_2_ normal rat groups were fed diets containing bael leaf extract 125 mg and 250 mg, respectively for consecutive 60 days. In a similar way, hyperlipidemic rats group G_h0_ was administrated basal diet while G_h1_ and G_h2_ hyperlipidemic rat groups were fed diets containing bael leaf extract 125 mg and 250 mg, respectively for consecutive 60 days. The blood drawn on day 0, day 30 and day 60 was analyzed for serum parameters, such as total cholesterol, high-density lipoprotein cholesterol, low–density lipoprotein cholesterol, triglycerides concentration and free and ester cholesterol.

**Results:**

Bael leaf powder is a rich source of crude fiber (14.50 ± 0.10 g/100 g). Aqueous extract of bael leaf contains alkaloids (15.58 ± 0.05 mg/g), flavonoids (64.00 ± 0.05 mg/g), phenolics (30.34 ± 0.01 GAEmg/g). From the In vivo studies, the lowest weight gain was observed in group G_2_ and in G_h2_ as compared to control of both groups. The decrease in serum TC for G_1_–15.06%, G_2_–17.27% while in G_h1_–22.46% and G_h2–_34.82% after day 60, respectively. The maximum decrease was observed in group G_2_ (− 14.33%) and in G_h2_ (− 24.79%) for triglycerides after 60 days. For HDL-cholesterol, significant increase (11.20%) in G_2_ and (49.83%) in G_h2_ was observed of after 60 days. A trend in decrease of serum LDL–cholesterol in G_2_ (− 9.63%) and in G_h2_ (− 44.65%) was also observed at day 60, and − 19.05% and − 30.06% decrease was noted in G_2_ and G_h2_, respectively and decreasing trend was observed in free and total cholesterol − 22.30% and − 81.49% for groups G_2_ and G_h2_ after day 60.

**Conclusions:**

The results of the present study demonstrated that the extract contents of bael leaf provide protective role against hypercholesterolemic and hyperlipidemic conditions.

## Background

In recent times, focus on plant research has improved all over the world and increasing number of studies have demonstrated potential health benefits of medicinal plants used in numerous traditional systems. It has been shown that many parts of plants for example leaves, stems, roots, fruits, and seeds provide health and nutrition providing bioactive compounds in human diet [1]. The bael (*Aegle marmelos*) has enormous traditional uses in the treatment of chronic diarrhea, dysentery, peptic ulcers and as a laxative. Past studies depicted antifungal, antibacterial, antiprotozoal and hypoglycemic properties where-as its cardiac effect has been investigated effectively as bael leaf extract in lower doses increases both heart rate and amplitude of contraction while in higher doses transiently inhibit the heart, followed by further stimulation [2].

Bael is a slow growing medium sized tree, up to 15 m tall with short trunk, thick, flaking bark, sometimes spiny branches, the lower ones drooping [3]. The deciduous alternate leaves, singly, two or three, are composed of 3 to 5 oval, pointed leaflets 4 to 10 cm long, 2 to 5 cm wide, the terminal one with a long petiole [4]. Bael is known for its medicinal properties, especially, the leaves and fruits which has shown cardiac and circulatory stimulatory activities. Bael extract has protective effect against jaundice, also shown astringent and carminative activities. Bael leaf extract is also effective against thyroid related disorder and swelling of joints. It has also been reported to possess anti-diabetic, antimicrobial, antifungal, cardioprotective, antiulcer, antihyperlipidemic effects, and are commonly used for the treatment of different ailment in the indigenous system of medicine in the Asian sub-continent [5].

Different types of carotenoids have been reported in the bael, which are responsible for the imparting yellow pale colour to fruit. Minor constituents such as ascorbic acid, sitosterol, crude fiber, tannins, α- amyrin, carotenoids, and crude proteins are also present. More than 100 bioactive compounds have been isolated from different parts of this plant. Various phytochemicals that have been extracted from leaf part of this plant are Skimmianine, Aeglin, Rutin, γ-sitosterole, β-sitosterol, Flavone, Lupeol, Cineol, Citral, Glycoside, O-isopentenyl, Hallordiol, Mameline, Citronellal, Cuuminaldehyde phenylethyle cinnamamides, Euginol, Marmesinin, Aegelin, Glycoside have been shown to be biologically active against various major and minor diseases [6].

Bael leaf extract contains alkaloids, emodins, ferric chloride, lead acetate, gelatin, phenolics, and volatile oils which may exhibit potential anticholesterol activity. The plant materials have these useful properties, which can be used for numerous medicinal applications and can be used by pharmaceutical company. Hyperlipidemia is a serious health problem and greatest cause of death all over the world, and if not treated, it is responsible for many complications affecting various organs in the body [7]. Hyperlipidemia may be treated using antilipidemic drugs but the hyperlipidemia originating from diabetes, renal lipid nephrosis or hypothyroidism necessitates the treatment of the original disease rather than hyperlipidemia [8]. The findings of the study of Sinha et al. [9] revealed that ethanolic extracts of bael leaves can effectively control the blood cholesterol and triglycerides levels in dyslipidaemic conditions. Oral administration of aqueous extract of bael fruits, leaves and seeds separately to a dose of 250 mg/kg to streptozotocin induced diabetic rats significantly lowered the serum and tissue lipid profile [10, 11]. The main objectives of this study were characterization of bael leaf extract for its bioactive constituents, antihypercholestrolemic and antilipidemic perspectives.

## Methods

### Procurement of material

Fully ripe fresh bael (*Aegle marmelos* Linn.) leaves, without any visual defects, were collected from University of Agricultural Faisalabad, Pakistan. Bael leaves were washed by water to remove dust/debris and dried at 50 °C, followed by grinding to a coarse powder consistency [12] and stored at − 20 °C until used.

### Aqueous extraction

Bael leaves, after washing, thoroughly were hot air-dried for 3 days at room temperature (~ 25 ͦ C). The dried leaves were pulverized using pestle mortar to obtain a powdered form which was stored in airtight glass vials at 4 ͦ C until used. The powdered plant material was mixed with distilled water (1:5) and stirred overnight at room temperature. The residue was removed by filtration through Whatman no.1 filter paper and the aqueous extracts was lyophilized and stored in airtight glass vials [13]. All extracts were stored in sterilized containers at − 20 °C until used for testing.

All extraction and subsequent analyses were done using three replicates.

### Chemical composition

The powder of bael leaf was chemically estimated. Moisture content was analyzed by using air forced draft oven. The sample was dried at 105 ± 5 °C to constant weight and calculations was made [14] (AACC Method No. 44-15A). For determination of crude protein, nitrogen percentage was assessed through Kjeltech Apparatus. The protein was calculated by multiplying percent nitrogen with conversion factor, 6.25 [15] (AOAC Method No. 990.03). Crude fat was estimated in oven-dried sample by Soxtec System. Sample weighing 5 g was used for extraction of crude fat with petroleum ether. After extraction, residue was dried till constant weight [14] (AACC Method No. 30–10). After fat extraction, samples were examined for crude fiber through Labconco Fibertech. Two g of fat-free sample was digested firstly with 1.25% H_2_SO_4_ and finally with 1.25% NaOH for 30 min. The residue was dried and weighed followed by ignition at 550 ± 15^°^C and cooled for further calculations [15] (AOAC Method No. 978.10). For determination of ash content, sample was taken in pre-weighed crucible and charred on burner till no visible fumes before incineration in the Muffle Furnace to obtain white grayish residue with no other taints [14] (AACC Method No. 08–01).

### Phytochemical profiling

#### Determination of alkaloids

Alkaloids content were measured by following the method of Harborne [16]. A suspension was prepared by dispersing 5 g of the dried leaves in 10% acetic acid solution in ethanol and kept at 28 °C for 4 h which was further filtered through Whatman number. 42. Thereafter, alkaloid was precipitated by concentrating the filtrate to one-fourth of its original volume and drops of conc. Aqueous NH_4_OH were added. Finally, the precipitate was washed with 1% ammonia solution and dried at 80 °C in the oven. The content of alkaloid was calculated and expressed as mg/g dry weight basis.

#### Determination of flavonoids

The flavonoids content were determined by Harborne method [16]. Briefly, 5 g of leaves was boiled in 2 M HCl for 30 min under reflux and filtered after cooling down to room temperature. Equal volume of ethyl acetate was then added drop-wise to the filtrate. The weight of precipitated flavonoids was determined and reported as mg/g dry weight basis.

#### Total phenolic content (TPC)

TPC of extracts were determined using Folin–Ciocalteu’s reagent method [17]. Briefly, 100 μl of different aqueous extracts of bael leaf, 500 μl of Folin–Ciocalteu’s reagent and 1 ml sodium carbonate were added and incubated at ambient temperature (25–27^°^C) for 90 min. The developed color was measured at 760 nm using UV–VIS spectrophotometer (UV-2450 PC, Shimadzu, Japan). Total phenolic content (TPC) were expressed as gallic acid equivalent (GAE mg/ g of dry mass.

### Efficacy studies

#### Experimental animals and diets

The study program was designed after the review and approval of ethical guidelines set by parent institute. Male albino rats (age 80–90 days, body weight 170 ± 10 g) were purchased from the National Institute of Health, Islamabad, Pakistan. The rats were individually housed in stainless steel wire-bottom cages in an environmentally controlled room at 25 ± 2 °C and 60 ± 5% relative humidity with a 12 h light–dark cycle. All groups of rat received basal diet consisting of 65% starch, 10% casein, 10% corn oil, 3% salt mixture, 1% vitamins mixture and 10% cellulose and addition of 1% cholesterol for induction of hyperlipidemia. At the initiation of study, rats (*n* = 10) were slaughtered to establish base line serum parameters. Hyperlipidemia was confirmed by measuring the levels of serum lipids and lipoproteins in the rats. Afterwards, normal rats group G_0_ was administrated basal diet while G_1_ and G_2_ normal rat groups were fed diets containing bael leaf extract 125 mg and 250 mg, respectively for consecutive sixty days. In a similar way, hyperlipidemic rats group G_h0_ was administrated basal diet while G_h1_ and G_h2_ hyperlipidemic rat groups were fed diets containing bael leaf extract 125 mg and 250 mg, respectively for consecutive sixty days. Each group was comprised of 10 rat animals. The amount of diet consumed by each rat was determined by deducting the left-over and spilled diet from the total amount supplied per day [18]. Gain in body weight of individual rats was determined after 30 and 60 day intervals.

#### Serum collection

At day 0, 30 and 60, Blood samples were collected through cardiac puncture, and serum was collected following the method of Uchida et al. [19].

### Biochemical profile

#### Serum lipid profile

Serum cholesterol level was determined using CHOD–PAP method described by Stockbridge et al. [20]. High density lipoprotein (HDL) in serum samples was measured by HDL cholesterol precipitant method described by Assmann [21]. Serum samples were also analyzed for low density lipoproteins (LDL) following the procedure of McNamara et al. [22]. Total triglycerides in all serum samples were determined by liquid triglycerides (GPO–PAP) method as outlined by Annoni et al. [23].

#### Estimation of free and ester cholesterol

To 0.2 ml of serum homogenate in a test tube, 3 ml of acetone ethanol mixture was added and kept on a water bath to raise its temperature to 95 °C. This mixture was vortexed for 15 min and the precipitated protein was separated by centrifugation. The protein precipitate was washed again with 3 ml of acetone ethanol mixture and the supernatants were pooled. 1 ml of glacial acetic acid was added, followed by a drop of 10% glacial acetic acid and the contents were mixed well.

The tubes were securely closed and kept in a dark chamber for 16 h. The precipitated cholesterol digitonide was removed by centrifugation at 1000 x *g* for 15 min. The precipitate was then dissolved in 10 ml of glacial acetic acid by heating over in a water bath. To this 1 ml of isopropanol was added followed by 2 ml of 0.1% ferric chloride solution and mixed well. After 5 min, 2 ml of concentrated H_2_SO_4_ was added drop-wise with constant shaking. The colour developed was read at 540 nm along with a standard cholesterol solution [24]. The esterified cholesterol was calculated from the difference between total and free cholesterol.

### Statistical analysis

The average of the three samples was reported as the measured value with standard deviation. The Duncan’s multiple range (DMR) test was used to estimate the level of significance that existed between the mean values for data analysis. The sample analysis for biochemical profile and study day intervals was carried out in triplicate and the significant differences were calculated among means at a probability level of 5% using Minitab version 14.1 [25].

## Results

Bael leaf power contained moisture content 6.80 ± 0.40 g/100 g, crude protein 6.00 ± 0.10 g/100 g, crude fat 1.95 ± 0.10 g/100 g, crude fiber 14.50 ± 0.10 g/100 g and ash 9.40 ± 0.10 g/100 g. The quantitative phytochemical estimation showed that the leaf contained a significant amount of alkaloids, flavonoids and phenolics. The mean values for these phytochemicals were: alkaloids (15.58 ± 0.05 mg/g), flavonoids (64.0 ± 0.05 mg/g) and phenolics (30.34 ± 0.01 mg/g).

**Fig. 1:**
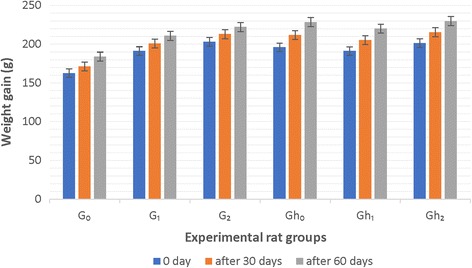
Weight gain of various rat groups in 30 and 60 days of study trial (G_0_: Control Normal rat group. G_1_: Normal rat group fed on 125 mg bael leaf extract. G_2_: Normal rat group fed on 250 mg bael leaf extract. Gh_0_: Control Hyperlipidemic rat group. Gh_1_: Hyperlipidemic rat group fed on 125 mg bael leaf extract. Gh_2_: Hyperlipidemic rat group fed on 250 mg bael leaf extract)

There was a significant decrease in body weight of hyperlipidemic group of rats with respect to normal group (Fig. 1). For normal group of rats, highest weight gain was observed in group G_0_ followed by G_1_, while the lowest increase was in group G_2_ after 30 days. Same trend was recorded after 60-day for normal group of rats. For hyperlipidemic group, the highest weight gain was observed in group G_h0_, whereas, the lowest weight gain was noted in G_h2_ after 30 days. At the end of 60-day the highest weight gain was noted in group G_h0_ and the lowest in G_h2_ followed by G_h1_.

Lipid profile of normal and hyperlipidemic group rats was observed after 30 and 60 day of study trial (Table 1). After 30 days, the highest significant decrease of ˗8.88% was observed in group G_2_ in normal rats whereas a significant decrease of ˗8.84% was noted in group G_1_. After 60 days, highly significant decrease was noted in group G_2_ (− 17.27%) and in group G_1_ (− 15.06%) in normal rats. In hyperlipidemic rat group, significant decrease was observed in rat groups. The decreasing trend was noted in G_h1_ (− 11.91%) but lesser than G_h2_ (− 17.49%) after 30-day trial as compared to the control group. After 60 days, the cholesterol level was decreased significantly in G_h1_ (− 22.46%) and G_h2_ (− 34.82%) groups, respectively than values taken as base line.

**Table 1:** Cholesterol of various groups of rats in 60 days study trial

Rat Group	Cholesterol (mg/dL)		
	Day-0	Day-30	Day-60
Control			
G_0_	96.56 ± 2.43^Aa^	97.13 ± 2.52^Aa^	97.01 ± 2.78^Aa^
G_1_	95.74 ± 1.85^Aa^	87.27 ± 1.76^Bb^	81.32 ± 1.28^Bc^
G_2_	97.11 ± 1.71^Aa^	88.48 ± 1.39^Bb^	80.33 ± 1.49^Bc^
Net Changes in Control Group			
G_1 -_ G_0_	0.82 ± 0.58^c^	9.86 ± 0.76^b^	15.69 ± 1.50^a^
G_2 -_ G_0_	0.55 ± 0.72^c^	8.65 ± 1.13^b^	16.68 ± 1.29^a^
G_2 -_ G_1_	1.37 ± 0.14^c^	1.21 ± 0.37^c^	0.99 ± 0.21^c^
HPL Group			
G_h0_	194.86 ± 2.93^Ca^	240.34 ± 2.48^Ba^	279.42 ± 2.26^Aa^
G_h1_	193.94 ± 3.81^Aa^	170.84 ± 2.21^Bb^	150.37 ± 2.08^Bc^
G_h2_	195.50 ± 3.74^Aa^	161.29 ± 2.98^Bc^	127.41 ± 2.35^Cc^
Net Changes in HPL Group			
G_h1 -_ G_h0_	0.92 ± 0.88^g^	69.50 ± 0.58^d^	129.05 ± 0.18^b^
G_h2 -_ G_h0_	0.64 ± 0.81^g^	79.05 ± 0.50^c^	152.01 ± 0.09^a^
G_h2 -_ G_h1_	1.56 ± 0.07^g^	9.55 ± 0.77^f^	22.96 ± 0.27^e^

Mean ± Standard Deviation; *n* = 10

G_0_: Control Normal rat group. G_1_: Normal rat group fed on 125 mg

bael leaf extract. G_2_: Normal rat group fed on 250 mg bael leaf extract

G_h0_: Control Hyperlipidemic rat group. G_h1_: Hyperlipidemic rat group fed on 125 mg

bael leaf extract. G_h2_: Hyperlipidemic rat group fed on 250 mg bael leaf extract

HPL: Hyperlipidemic group of rats

Means showing different lower case letters (a, b, c, d, e, f, g) represent significant differences (*p* < 0.05) within control or HPL groups. Whereas, means showing different upper case letters (A, B, C) represent significant differences (*p* < 0.05) across day-0 through day-60

A significantly lower triglycerides level was observed in normal groups G_1_ and G_2_ as compared to G_0_ after 30 days (Table 2). In 60-day trial, the normal group of rats showed significant decrease in G_1_ (rats fed on 125 mg bael leaf extract) and G_2_ (rats fed on 250 mg bael leaf extract) groups. However, triglyceride level for hyperlipidemic group was decreased in G_h1_ (˗11.99%) and G_h2_ (− 14.01%) as compared to values taken as base line in first 30 days. After 60 days the highest decrease was noted in G_h2_ (− 24.79%) than G_h1_ (− 21.84%).

**Table 2:** Triglycerides of various groups of rats in 60 days study trial

Rat Groups	Triglycerides (mg/dL)		
	Day-0	Day-30	Day-60
Control			
G_0_	101.61 ± 1.28^Bb^	102.74 ± 1.56b^AB^	104.08 ± 1.72^Aa^
G_1_	118.47 ± 2.00^Aa^	110.35 ± 1.85^aB^	103.75 ± 2.48^Ac^
G_2_	93.98 ± 2.11^Ac^	82.86 ± 2.04^cB^	72.91 ± 2.69^Bc^
Net Changes in Control Group			
G_1 -_ G_0_	16.86 ± 0.72^e^	7.61 ± 0.29^f^	0.33 ± 0.76^g^
G_2 -_ G_0_	7.63 ± 0.83^f^	19.88 ± 0.48^d^	31.17 ± 0.97^a^
G_2 -_ G_1_	24.49 ± 0.11^c^	27.49 ± 0.19^b^	30.84 ± 0.21^a^
HPL Group			
G_h0_	145.17 ± 3.69^Cb^	162.31 ± 3.70^Bb^	194.12 ± 3.33^Aa^
G_h1_	161.10 ± 3.74^Aa^	141.77 ± 3.44^Ba^	125.91 ± 3.65^Bc^
G_h2_	160.59 ± 2.62^Aa^	138.09 ± 2.40^Ba^	120.77 ± 2.34^Cc^
Net Changes in HPL Group			
G_h1 -_ G_h0_	15.93 ± 0.05^e^	20.54 ± 0.26^d^	68.21 ± 0.32^b^
G_h2 -_ G_h0_	15.42 ± 1.07^e^	24.22 ± 1.30^c^	73.35 ± 0.99^a^
G_h2 -_ G_h1_	0.51 ± 1.12^h^	3.68 ± 1.04^g^	5.14 ± 1.31^f^

Mean ± Standard Deviation; *n* = 10

G_0_: Control Normal rat group. G_1_: Normal rat group fed on 125 mg

bael leaf extract. G_2_: Normal rat group fed on 250 mg bael leaf extract

G_h0_: Control Hyperlipidemic rat group. G_h1_: Hyperlipidemic rat group fed on 125 mg

bael leaf extract. G_h2_: Hyperlipidemic rat group fed on 250 mg bael leaf extract

HPL: Hyperlipidemic group of rats

Means showing different lower case letters (a, b, c, d, e, f, g, h) represent significant differences (*p* < 0.05) within control or HPL groups. Whereas, means showing different upper case letters (A, B, C) represent significant differences (*p* < 0.05) across day-0 through day-60

HDL cholesterol level showed significant differences in the normal and hyperlipidemic groups of rats. After 30 days, there was no specific change was seen in normal group of rats as compared to the values taken as base line. After 60 days, the increasing trend was noted in G_2_ than G_1_ as compared to control group. For hyperlipidemic group of rats, the highest increase was noted in G_h2_ than G_h1_ after 30 days. After 60-days, the maximum increasing trend was seen in G_h2_ than G_h1_, respectively (Table 3).

**Table 3:** HDL of various groups of rats in 60 days study trial

Rat Groups	HDL (mg/dL)		
	Day-0	Day-30	Day-60
Control			
G_0_	49.53 ± 0.36^Bb^	52.89 ± 0.44^Aba^	51.05 ± 0.24^Ba^
G_1_	50.60 ± 0.35^Aa^	51.91 ± 0.25^Aa^	53.07 ± 0.31^Aab^
G_2_	49.07 ± 0.67^Ba^	51.71 ± 0.89^Ba^	54.48 ± 0.79^Aa^
Net Changes in Control Group			
G_1 -_ G_0_	1.07 ± 0.01^f^	0.98 ± 0.19^f^	2.02 ± 0.07^b^
G_2 -_ G_0_	0.46 ± 0.31^g^	1.18 ± 0.45^e^	3.43 ± 0.55^a^
G_2 -_ G_1_	1.53 ± 0.32^c^	0.20 ± 0.64^h^	1.41 ± 0.48^d^
HPL Group			
G_h0_	30.01 ± 0.27^Aa^	28.70 ± 0.84^ABb^	26.77 ± 0.27^Cb^
G_h1_	30.12 ± 0.31^Ca^	36.82 ± 0.98^Ba^	40.18 ± 0.64^Ba^
G_h2_	30.34 ± 0.76^Ca^	38.39 ± 0.63^Ba^	45.46 ± 0.90^Aa^
Net Changes in HPL Group			
G_h1 -_ G_h0_	0.11 ± 0.04^g^	8.12 ± 0.14^d^	13.41 ± 0.37^b^
G_h2 -_ G_h0_	0.33 ± 0.49^g^	9.69 ± 0.21^c^	18.69 ± 0.63^a^
G_h2 -_ G_h1_	0.22 ± 0.45^g^	1.57 ± 0.35^f^	5.28 ± 0.26^e^

Mean ± Standard Deviation; *n* = 10

G_0_: Control Normal rat group. G_1_: Normal rat group fed on 125 mg

bael leaf extract. G_2_: Normal rat group fed on 250 mg bael leaf extract

G_h0_: Control Hyperlipidemic rat group. G_h1_: Hyperlipidemic rat group fed on 125 mg

bael leaf extract. G_h2_: Hyperlipidemic rat group fed on 250 mg bael leaf extract

HPL: Hyperlipidemic group of rats

Means showing different lower case letters (a, b, c, d, e, f, g) represent significant differences (*p* < 0.05) within control or HPL groups. Whereas, means showing different upper case letters (A, B, C) represent significant differences (*p* < 0.05) across day-0 through day-60

The LDL was increased from normal level after giving basal diet along with additional cholesterol (Table 4), however, after administration of bael leaf extract it showed a significant decrease in LDL level of rats. Decrease was noted in normal group (G_1_ and G_2_) after 30 days (− 2.44% to − 4.59%) and 60 days (− 4.96% to − 9.63%) of study according to the values taken as base line whereas the decrease was noted after 30 to 60 day while the decrease was higher in hyperlipidemic group (G_h1_ and G_h2_) –16.06% and − 20.15% after ay 30. After 60 days the LDL values had dropped down close to normal levels.

**Table 4:** LDL of various groups of rats in 60 days study trial

Rat Groups	LDL (mg/dL)		
	Day-0	Day-30	Day-60
Control			
G_0_	134.10 ± 2.13^Ab^	134.24 ± 2.44^aB^	134.24 ± 2.40^Aa^
G_1_	141.69 ± 2.72^Aa^	138.23 ± 2.5^Ab^	134.66 ± 2.35^Ac^
G_2_	138.71 ± 2.19^Aa^	132.34 ± 2.55^Bb^	125.34 ± 2.20^Bc^
Net Changes in Control Group			
G_1 -_ G_0_	7.59 ± 0.59^b^	3.99 ± 0.06^e^	0.42 ± 0.05^h^
G_2 -_ G_0_	4.61 ± 0.06^d^	1.90 ± 0.11^g^	8.90 ± 0.20^a^
G_2 -_ G_1_	2.98 ± 0.53^f^	5.89 ± 0.05^c^	9.32 ± 0.15^a^
HPL Group			
G_h0_	140.07 ± 2.70^aC^	164.55 ± 2.79^Ab^	186.68 ± 3.39^Aa^
G_h1_	143.25 ± 2.05^aA^	120.24 ± 2.15^Bb^	95.56 ± 1.34^Bc^
G_h2_	143.33 ± 2.13^aA^	114.44 ± 2.11^Cb^	79.32 ± 1.98^Cc^
Net Changes in HPL Group			
G_h1 -_ G_h0_	3.18 ± 0.65^g^	44.31 ± 0.64^d^	91.12 ± 2.05^b^
G_h2 -_ G_h0_	3.26 ± 0.57^g^	50.11 ± 2.11^c^	107.36 ± 1.41^a^
G_h2 -_ G_h1_	0.08 ± 0.07^h^	5.80 ± 0.04^f^	16.24 ± 0.64^e^

Mean ± Standard Deviation; *n* = 10

G_0_: Control Normal rat group. G_1_: Normal rat group fed on 125 mg

bael leaf extract. G_2_: Normal rat group fed on 250 mg bael leaf extract

G_h0_: Control Hyperlipidemic rat group. G_h1_: Hyperlipidemic rat group fed on 125 mg

bael leaf extract. G_h2_: Hyperlipidemic rat group fed on 250 mg bael leaf extract

HPL: Hyperlipidemic group of rats

Means showing different lower case letters (a, b, c, d, e, f, g, h) represent significant differences (*p* < 0.05) within control or HPL groups. Whereas, means showing different upper case letters (A, B, C) represent significant differences (*p* < 0.05) across day-0 through day-60

Free cholesterol was elevated in cholesterol fed rats as compared to the normal group. But in normal group of rats, significant decrease was noted in G_1_ and G_2_ groups as compared to the control after 30 days, this decrease was two-fold higher in these groups after 60 days. Whereas, in the hyperlipidemic group of rats, elevated level of free cholesterols turned to a normal range after 60 days as a result of bael leaf extract in their diet (Table 5).

**Table 5:** Free Cholesterol of various groups of rats in 60 days study trial

Rat Groups	Free Cholesterol (mg/dL)		
	Day-0	Day-30	Day-60
Control			
G_0_	20.34 ± 0.13^Bb^	21.29 ± 0.44^Aa^	21.88 ± 0.40^Aa^
G_1_	22.02 ± 0.72^Aa^	20.20 ± 0.23^Bb^	18.38 ± 0.30^Bc^
G_2_	21.52 ± 0.95^Aab^	19.38 ± 0.38^Cb^	17.42 ± 0.48^Cc^
Net Changes in Control Group			
G_1 -_ G_0_	1.68 ± 0.59^d^	1.09 ± 0.21^f^	3.50 ± 0.10^b^
G_2 -_ G_0_	1.18 ± 0.82^e^	1.91 ± 0.06^c^	4.46 ± 0.08^a^
G_2 -_ G_1_	0.5 ± 0.23^i^	0.82 ± 0.15^h^	0.96 ± 0.18^g^
HPL Group			
G_h0_	24.51 ± 1.38^Cb^	27.08 ± 0.96^Ab^	30.21 ± 0.76^Aa^
G_h1_	27.00 ± 1.53^Aa^	23.02 ± 1.46^Bb^	20.34 ± 1.51^Bc^
G_h2_	25.81 ± 0.91^Aa^	22.22 ± 1.33^Bab^	18.93 ± 1.06^Bc^
Net Changes in HPL Group			
G_h1 -_ G_h0_	2.49 ± 0.15^e^	4.06 ± 0.50^d^	9.87 ± 0.75^b^
G_h2 -_ G_h0_	1.30 ± 0.47^f^	4.86 ± 0.37^c^	11.28 ± 0.30^a^
G_h2 -_ G_h1_	1.19 ± 0.62^f^	0.80 ± 0.13^g^	1.41 ± 0.45^f^

Mean ± Standard Deviation; *n* = 10

G_0_: Control Normal rat group. G_1_: Normal rat group fed on 125 mg

bael leaf extract. G_2_: Normal rat group fed on 250 mg bael leaf extract

G_h0_: Control Hyperlipidemic rat group. G_h1_: Hyperlipidemic rat group fed on 125 mg

bael leaf extract. G_h2_: Hyperlipidemic rat group fed on 250 mg bael leaf extract

HPL: Hyperlipidemic group of rats

Means showing different lower case letters (a, b, c, d, e, f, g, h, i) represent significant differences (*p* < 0.05) within control or HPL groups. Whereas, means showing different upper case letters (A, B, C) represent significant differences (*p* < 0.05) across day-0 through day-60

A significant change for ester cholesterol was observed after 60 days in normal rats as compared to the values taken as base line (Table 6). The decrease was (− 9.6%) and (− 11.52%) in groups G_1_ and G_2_ after 30 days while the decrease was (− 22%) and (− 22.30%) after 60 days of study. In hyperlipidemic rats, the significant reduction was recorded after 60 days. After 30 days rats, significant decrease, − 18.46% and − 34.26%, was observed in groups G_h1_ and G_h2_ respectively. A decrease of − 34.94% and − 59.89% was seen in G_h1_ andG_h2_, respectively.

**Table 6:** Ester Cholesterol of various groups of rats in 60 days study trial

Rat Groups	Ester Cholesterol (mg/dL)		
	Day-0	Day-30	Day-60
Control			
G_0_	2.20 ± 0.06^Bc^	2.46 ± 0.25^aAB^	2.60 ± 0.18^Aa^
G_1_	2.50 ± 0.15^Ab^	2.26 ± 0.19^Ab^	1.95 ± 0.08^Bc^
G_2_	2.69 ± 0.19^Aa^	2.38 ± 0.38^Ab^	2.09 ± 0.12^Bc^
Net Changes in Control Group			
G_1 -_ G_0_	0.30 ± 0.09^c^	0.20 ± 0.06^d^	0.65 ± 0.10^a^
G_2 -_ G_0_	0.49 ± 0.13^b^	0.08 ± 0.13^g^	0.51 ± 0.06^b^
G_2 -_ G_1_	0.19 ± 0.04^e^	0.12 ± 0.19^f^	0.14 ± 0.04^f^
HPL Group			
G_h0_	3.55 ± 0.11^Cb^	5.80 ± 0.16^Ab^	8.47 ± 0.26^Aa^
G_h1_	3.52 ± 0.23^Aa^	2.87 ± 0.51^Ab^	2.29 ± 0.32^Bc^
G_h2_	3.94 ± 0.35^Aa^	2.59 ± 0.35^Bb^	1.58 ± 0.15^Cc^
Net Changes in HPL Group			
G_h1 -_ G_h0_	0.03 ± 0.12^h^	2.93 ± 0.35^d^	6.18 ± 0.06^b^
G_h2 -_ G_h0_	0.39 ± 0.24^f^	3.21 ± 0.19^c^	6.89 ± 0.11^a^
G_h2 -_ G_h1_	0.42 ± 0.12^f^	0.28 ± 0.16^g^	0.71 ± 0.17^e^

Mean ± Standard Deviation; *n* = 10

G_0_: Control Normal rat group. G_1_: Normal rat group fed on 125 mg

bael leaf extract. G_2_: Normal rat group fed on 250 mg bael leaf extract

G_h0_: Control Hyperlipidemic rat group. G_h1_: Hyperlipidemic rat group fed on 125 mg

bael leaf extract. G_h2_: Hyperlipidemic rat group fed on 250 mg bael leaf extract

HPL: Hyperlipidemic group of rats

Means showing different lower case letters (a, b, c, d, e, f, g, h) represent significant differences (*p* < 0.05) within control or HPL groups. Whereas, means showing different upper case letters (A, B, C) represent significant differences (*p* < 0.05) across day-0 through day-60

## Discussion

Various studies have been done to analyze the proximate composition of the bael parts, It was found that bael leaf, pulp and seed powder were good source of protein, fat, minerals, crude fiber and energy rich source of available carbohydrates, dietary fiber and also contain antinutrients, which help in controlling cholesterol. Another study reported that bael leaf powder had 10.30 g ash [26]. The proximate composition of wild variety of bael leaves (Gir Forest) and cultivated variety (Gomayasi) Gujarat, India) were studied and it was found that both varieties had good nutritional components, however, the wild variety was superior to the cultivated variety in nutrients, such as carbohydrates, protein, fiber, moisture, ash content [27].

Phytochemical screening of ethanolic extract of bael leaf has been shown to reveal the presence of alkaloids [28]. The alkaloids content was quantitatively estimated and was found in the range of 3.78 ± 0.15 to 16.08 ± 0.05 mg/g in different varieties of bael leaves [29]. Similarly, all the varieties exhibited good quantity of flavonoids and phenols starting from 10.40 ± 0.047 mg/mL and 5.80 ± 0.085 mg/mL, respectively [30].

According to the findings of Muralidharan et al. [31] no significant body weight changes were found in rats fed on bael leaf extract. Increase in body weight of rats fed on basal diet in 60-day trial was normal [32]. Umbelliferone (a benzopyrone present in Bael) possesses antihyperlipidemic effect and decreased the level of total cholesterol, very low-density lipoprotein cholesterol, LDL cholesterol, triglycerides, free fatty acids and phospholipids in the plasma, liver and kidneys of diabetic rats. It also increased the levels of HDL in plasma of rats [33]. This activity of bael leaf may maintain the body weight near to normal level.

Bael leaves are reported to contain coumarines, umbelliferone and esculetin which can increase the process of lipolysis [34]. The levels of serum total cholesterol and triglycerides were found to be significantly reduced in the bael extract treated diabetic animals. This might be due to the reduced hepatic triglyceride synthesis and or reduced lipolysis bael extract treated rats. The HDL levels increased significantly in the bael extract treated rats indicating a reversed atherogenic risk [35].

The lipid lowering activity of bael leaf extract may be attributed to the phytoconstituents present such as saponins and phenolic ingredients present in it as reported for other plant extracts [36]. Umbelliferone (a benzopyrone present in bael) possesses antihyperlipidemic properties and decreased the level of total cholesterol, very low-density lipoprotein cholesterol, LDL cholesterol, triglycerides, free fatty acids and phospholipids in the plasma, liver and kidneys of diabetic rats. It also increased the levels of HDL in plasma of rats, which can also maintain the body weight closer to normal level [33].

In a study conducted by Suriyamoorthy et al. [7], there was a significant increase in the free cholesterol (66.5%) and ester cholesterol levels (535.24%) in oil induced group compared to that of the normal group. Treatment with herbal drugs like bael showed a significant decrease in the free cholesterol (− 17.65%) and ester cholesterol levels (− 59.87%). Free and ester cholesterol reduced significantly on treating the HFD rats with methanolic extract of Ipomoea digitata [37] and this supports the antioxidant activity of this herb. In our experiment we also got similar results that the ester cholesterol and free cholesterol were significantly increased in the cholesterol induced rats when compared to control. This elevated level of cholesterol was due to the deposition of hepatic cholesterol as a result of administration of cholesterol. Upon treatment with the bael, the cholesterol level was reduced back to near normal level. It was also evident that the treatment with the extract of bael leaf inhibited the significant elevation of total cholesterol as compared to that of dyslipidemic animals [38].

## Conclusions

It is quite evident from this study that bael contains a number of phytoconstituents which reveals its uses for various therapeutic purposes against hyperlipidemia like metabolic syndrome. Bael leaf extract supplementation decreased total cholesterol, total triglyceride, LDL, HDL, free and ester cholesterol. Therefore, we suggest that active components of bael have a significant anti-hyperlipidemic effect. Limitations of this study are that the investigation was performed only on male rats, therefore, we cannot exclude the possibility that sex influences the effect of bael leaf extract on hypercholesterolemia or hyperlipidemia and survival mechanisms. Still, so much work is required with the bael to investigate the mechanism of actions with other therapeutic activities.
